# Muscle loss 6 months after surgery predicts poor survival of patients with non-metastatic colorectal cancer

**DOI:** 10.3389/fnut.2022.1047029

**Published:** 2022-12-01

**Authors:** Liang Zhang, Junjie Guan, Chao Ding, Min Feng, Longbo Gong, Wenxian Guan

**Affiliations:** ^1^Department of Gastrointestinal Surgery, Xuzhou Central Hospital, Xuzhou, China; ^2^Department of General Surgery, Drum Tower Clinical Medical College of Nanjing Medical University, Nanjing, Jiangsu, China; ^3^Department of General Surgery, Jiangsu Province Hospital of Chinese Medicine, Affiliated Hospital of Nanjing University of Traditional Chinese Medicine, Nanjing, Jiangsu, China

**Keywords:** colorectal cancer, sarcopenia, skeletal muscle loss, visceral adipose tissue, survival

## Abstract

**Background:**

Muscle loss is a common characteristic of cancer-related malnutrition and a predictor of poorer prognosis in oncological patients. This study evaluated the association between altered body composition 6 months after surgery and the prognosis in patients with non-metastatic colorectal cancer.

**Materials and methods:**

A total of 314 patients who underwent elective curative surgery were enrolled in the study. The third lumbar CT images on preoperative and 6-months postoperative were collected to calculate the skeletal muscle index (SMI), visceral adiposity index (VATI), and subcutaneous adiposity index (SATI). Sarcopenia was defined by the cut-off values reported in the literature, and risk factors affecting overall survival (OS) and disease-free survival (DFS) in CRC were analyzed using Cox regression models.

**Results:**

Eighty-two of 314 patients (26.1%) with CRC were diagnosed with sarcopenia before surgery, the preoperative sarcopenia was not significantly associated with the prognosis of CRC patients. There were significant differences in frequency of complications between patient groups according to sarcopenia (41.5 vs. 21.4%, *p* = 0.004). The Postoperative LOS (11.21 ± 3.04 vs. 8.92 ± 2.84, *p* < 0.001) was longer in the sarcopenia group than in the non-sarcopenia group, and 30-d readmission (24.4 vs. 6.0%, *p* < 0.001) was higher in the sarcopenia group compared to the non-sarcopenia group. In multivariate analysis, 6-months SMI loss > 10% after surgery was independently associated with poorer OS [hazard ratio (HR) = 3.74; 95% confidence interval (CI) 1.96 to 7.12; *P* < 0.001] and DFS (HR = 3.33; 95% CI, 1.71 to 6.47; *P* < 0.001). SMI changes were moderately correlated with changes in body mass index (BMI) (*R* = 0.47, *P* < 0.001).

**Conclusion:**

6-months muscle loss after surgery may affect overall and disease-free survival and was an independent predictor of prognosis in patients with CRC.

## Introduction

Colorectal cancer (CRC) is one of the common malignancies of the gastrointestinal tract in China, with the third-highest incidence and the fifth-highest mortality rates among all cancers in China (1). Compared to the average population, the incidence of malnutrition in CRC patients is even higher at 40 to 80% (2). Cancer cachexia is defined as body weight loss of ≥ 5% during the previous 6 months, or ≥ 2% if body mass index < 20 kg/m^2^ (3). It’s a disease characterized by weight loss, specifically loss of skeletal muscle and adipose tissue, which may lead to weight loss and sarcopenia. Adipose tissue is strongly associated with the development of CRC, and obesity not only increases the risk of CRC but has also been shown to be an independent risk factor for CRC prognosis (4). In addition, some researchers have concluded that the presence of preoperative sarcopenia affects the prognosis of many malignancies, including CRC (5), but some studies (6, 7) have contradicted this finding and thus remains doubtful. This may be due to the fact that most of the previous studies were based on the body composition of the patients before treatment. However, the body composition in patients with malignancy is dynamic and skeletal muscle and adipose tissue may increase or decline as the disease progresses or treatment is administered. Therefore, it is worth considering whether body composition observations at a particular period are sufficiently descriptive or representative of predicting patient outcomes. However, there is a lack of data on the potential impact of changes in skeletal muscle and adipose tissue during treatment on the prognosis of CRC patients.

In terms of methods to assess the nutritional status of patients, studies have shown that the cross-sectional areas of skeletal muscle and adipose tissue at the level of the third lumbar vertebra (L3) on abdominal computed tomography (CT) are strongly correlated with the total body skeletal muscle and fat masses, and that CT images can provide objective qualitative and quantitative measurements of the patient’s body composition (8, 9). CT images are widely used in the diagnostic examination, radiotherapy (RT) planning and long-term follow-up of CRC patients; clinicians can easily access body composition change during treatment. The ease of use and safety of the method and no additional expenses to the patient has made CT-based measurement of body composition one of the most popular research methods in recent years.

We hypothesized that sarcopenia, skeletal muscle loss, and adipose tissue change during treatment would affect patient outcomes. Therefore, This study collected abdominal CT images data from CRC patients before and after surgery to assess the impact of skeletal muscle and adipose tissue changes on clinical outcomes in CRC patients.

## Patients and methods

We retrospectively analyzed a total of 514 patients with CRC who underwent surgical resection with curative intent at Xuzhou Central Hospital from January 2015 and May 2017. Patients were excluded if they were died within 6 months after surgery (*n* = 21), did not have a preoperative or postoperative CT scan (*n* = 81), or if they had metastatic disease (*n* = 26), or missing visit (72). The final sample size was 314 patients. The study was conducted after review and approval by the Ethics Committee of Xuzhou Central Hospital.

The same board-certified colorectal surgeons treated all patients, and all enrolled patients underwent radical surgery. We obtained data regarding patients’ sex, age, height, weight, pathological TNM stage, and CT images from medical records. These were used to calculate BMI and body composition. A routine preoperative CT image was obtained before surgery, and a postoperative CT image was obtained close to 6 months after surgery.

### Body composition measurement and data collection

The third lumbar (L3) vertebra was selected as a standardized landmark. Preoperative and postoperative CT scans were extracted from each patient. Each image was segmented in MATLAB software for analysis. Skeletal muscle area in this plane was calculated by using Hounsfield unit (HU) thresholds of −29 and + 150, the subcutaneous fat area was calculated from extra muscular tissue with a density between −190 and −30 HU and visceral adipose tissue from non-subcutaneous tissue with a density between −150 and −50 HU. For assessment of inter-rater reliability, a random sample of 20 patients selected from this cohort was performed by two independent researchers. The intraobserver coefficients of variation were 0.6, 1.0, and 0.8% for the skeletal muscle area, and VAT area, SAT area respectively, which is regarded to be low. The cross-sectional skeletal muscle area (SMA), subcutaneous adipose tissue area (SATA), and visceral adipose tissue area (VATA) were measured in cm^2^ and normalized by the patient’s height (m^2^) to calculated indexes (cm^2^/m^2^) for skeletal muscle (SMI), subcutaneous adipose tissue (SATI), and visceral adipose tissue (VATI).

### Definitions of skeletal muscle index, subcutaneous adiposity index, and visceral adiposity index

The optimal cut-off values for SMI, SATI, and VATI have not been clearly defined, and in this study, sarcopenia was defined as an SMI of < 41.0 cm^2^/m^2^ according to the definition of Martin et al. (10). The cut-off values for SATI and VATI were set at the highest tertile for SATI and VATI as performed by other studies with similar population sizes (6, 11). We assessed the magnitude of change in skeletal muscle and adipose tissue before and after surgery, and patients with an increase or reduction in SMI, SATI, and VATI of > 10% were classified as having “SMI gain,” “SATI gain,” “VATI gain” or “SMI loss,” “SATI loss,” “VATI loss,” respectively.

### Outcome parameters

The primary endpoints of the study were OS and DFS. Overall survival was defined as the time from surgery to death from any cause for expired patients or the last follow-up for live patients. Disease-free survival was defined as the time from surgery to the time of recurrence. Secondary endpoints were postoperative complications (Clavien-Dindo Surgical Complication classification system) and hospital length of stay.

### Method of follow-up

Patients are followed up from the end of treatment until September 2020. The main components of the follow-up are: whether the patient is surviving, whether the tumor has recurred, their living status and whether they have any discomfort or complications arising from the treatment received. The duration of follow-up ranged from 0.6 to 6.75 years, with a median duration of 3.9 years.

### Statistical analysis methods

All data was statistically processed with R.4.1.0 software. The measurement data were expressed according to the type of data, with mean ± standard deviation if normally distributed and median and interquartile spacing when not normally distributed. The *t*-test was used for measurement data, the χ^2^ test was used to compare count data, and the rank-sum test was used for rank data. Survival curves were plotted using the Kaplan-Meier method, and differences were analyzed using the log-rank test (Log-Rank). Survival analyses were first performed using one-way analysis of variance. Single factors with *P* < 0.05 or substantiated by evidence were further included in Cox regression for multi-factor analysis. For testing correlations between BMI changes and body composition changes, Pearson correlation and one-way analysis of variance (ANOVA) were used, where appropriate. Pearson correlation factors of > 0.7 were considered a good correlation between datasets. A correlation was considered moderate at 0.4–0.7, and poor at < 0.4. The test level was set as a two-sided test, and differences were considered statistically significant at *P* < 0.05.

## Results

### Patient characteristics

In total, 314 patients with biopsy-proven AJCC stage I-III colorectal cancer who had received surgical resection with curative intent were enrolled. Clinical characteristics and perioperative outcomes according to the preoperative SMI category are summarized in Table 1. The mean age of all patients was 58.91 ± 11.48 years. Eighty-two patients (26.1%) had preoperative sarcopenia. The preoperative BMI (21.19 ± 2.81 vs. 24.16 ± 3.07, *p* < 0.001) were significantly lower in the sarcopenia group than in the non-sarcopenia group. Sarcopenia was noted more frequently in female patients (70.7 vs. 22.8%, *p* < 0.001) than in male patients.

**TABLE 1:** Clinical characteristics and perioperative outcomes according to the preoperative SMI category (*n* = 314).

	Overall (*n* = 314)	Sarcopenia (*n* = 82)	Non-sarcopenia (*n* = 232)	*p*-value
**Sex**				<0.001
Female	111 (35.4%)	58 (70.7%)	53 (22.8%)	
Male	203 (64.6%)	24 (29.3%)	179 (79.2%)	
**Age**	58.91 ± 11.48	60.87 ± 11.58	58.22 ± 11.55	0.07
**BMI**	23.36 ± 3.28	21.19 ± 2.81	24.16 ± 3.07	<0.001
**CRP (mg/L)**				0.11
**>10**	36 (11.5%)	14 (17.1%)	22 (9.5%)	
**<10**	262 (83.4%)	65 (79.3%)	197 (84.9%)	
**Missing**	16 (5.1%)	3 (3.6%)	13 (5.6%)	
**ALB (g/L)**				0.006
**>35**	301 (95.9%)	74 (90.2%)	227 (97.8%)	
**<35**	13 (4.1%)	8 (9.8%)	5 (2.1%)	
**ASA score**				0.63
I	178 (56.7%)	43 (52.4%)	135 (58.2%)	
II	102 (32.5%)	30 (36.6%)	72 (31.0%)	
III	34 (10.8%)	9 (11.0%)	25 (10.8%)	
**30-d Any complications**				0.004
No	224 (71.3%)	48 (58.5%)	176 (75.9%)	
YES	90 (28.7%)	34 (41.5%)	56 (24.1%)	
**30-d Major complications (Clavien Dindo score)**				
I-II	70 (22.2%)	27 (32.9%)	43 (18.5%)	0.66
III-IV	20 (6.4%)	6 (7.3%)	14 (6.0%)	
**Operation**				0.32
Right hemi-colectomy	28 (8.9%)	11 (13.4%)	17 (7.3%)	
LEFT hemi-colectomy	62 (19.7%)	12 (14.6%)	50 (21.6%)	
Dixon	204 (65.0%)	51 (62.2%)	153 (65.9%)	
Miles	20 (6.4%)	8 (9.8%)	12 (5.2%)	
**TNM stage**				0.341
I	46 (14.6%)	15 (18.3%)	31 (13.3%)	
II	150 (47.8%)	34 (41.5%)	116 (50.0%)	
III	118 (37.6%)	33 (40.2%)	85 (36.7%)	
**Neoadjuvant therapy afterpreoperative scan**				0.009
No	206 (95.1%)	53 (91.8%)	185 (96.8%)	
Yes	108 (4.9%)	29 (8.2%)	47 (3.2%)	
**Postoperative LOS, days**	9.71 ± 2.41	11.21 ± 3.04	8.92 ± 2.84	<0.001
**≤7**	165 (52.5%)	30 (36.6%)	135 (58.2%)	
**>7**	149 (47.5%)	52 (63.4%)	97 (41.8%)	
**30-d Readmission**				
No	280 (89.2%)	62 (75.6%)	218 (94.0%)	<0.001
Yes	34 (10.8%)	20 (24.4%)	14 (6.0%)	
**Incisional hernia**				1
No	298 (94.9%)	78 (95.1%)	220 (94.8%)	
Yes	16 (5.1%)	4 (4.9%)	12 (5.2%)	

### Body composition change during treatment

**FIGURE 1:**
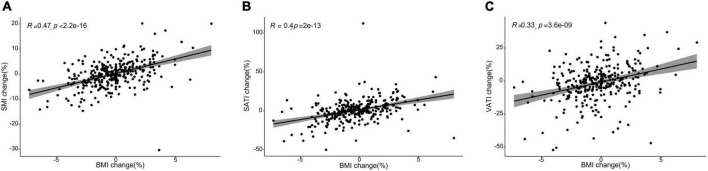
Scatter plots showing a correlation between the changes in body mass index (BMI) and body composition parameters from baseline to 6 months after treatment completion. **(A)** Skeletal muscle index (SMI) changes were moderately correlated with changes in body mass index (BMI) (*R* = 0.47, *P* < 0.001). **(B)** Subcutaneous adiposity index (SATI) changes were weakly correlated with changes in body mass index (BMI) (*R* = 0.4, *P* < 0.001). **(C)** Visceral adiposity index (VATI) changes were weakly correlated with changes in body mass index (BMI) (*R* = 0.33, *P* < 0.001).

The body composition changes during 6 months after surgery were summarized in Table 2. Forty-nine (15.6%), 213 (67.8%), and 52 (16.6%) patients were diagnosed with SMI loss, stable SMI, or SMI gain, respectively. VATI stable, VATI loss, VATI gain was seen in 96 (30.6%), 117 (37.3%), and 101 (32.1%) patients, respectively. SATI stable, SATI loss, SATI gain occurred in 108 (34.4%), 90 (28.7%), and 116 (36.9%) patients, respectively. The prevalence of SMI loss (19.0 vs. 6.1%, *p* < 0.05) was higher in patients with non-sarcopenia than in patients with sarcopenia. The prevalence of SATI and VATI changes were not significantly different between sarcopenia and non-sarcopenia groups. Patients in the sarcopenia group had a lower SMI than the non-sarcopenia group (36.58 ± 3.38 vs. 50.21 ± 7.07, *p* < 0.05), while VATI and SATI was higher in the non-sarcopenia group, with a statistically significant difference (43.21 ± 23.55 vs. 37.08 ± 21.80, *p* < 0.05; 43.93 ± 19.70 vs. 34.53 ± 17.93, *p* < 0.001). The changes in BMI were correlated to the changes in SMI (*R* = 0.47, *P* < 0.001), SATI (*R* = 0.4, *P* < 0.001), VATI (*R* = 0.33, *P* < 0.001) (Figure 1).

**TABLE 2:** The body composition changes during 6 months after surgery (*n* = 314).

	Overall (*n* = 314)	Sarcopenia (82)	Non-sarcopenia (232)	*p*-value
**SMI**	46.65 ± 8.71	36.58 ± 3.38	50.21 ± 7.07	<0.001
**SMI change**				0.0019
SMI stable (±10.0%)	213 (67.8%)	56 (68.3%)	157 (67.7%)	
SMI loss (> –10.0%)	49 (15.6%)	5 (6.1%)	44 (19.0%)	
SMI gain (> +10.0%)	52 (16.6%)	21 (25.7%)	31 (13.3%)	
**SATI**	41.48 ± 19.69	34.53 ± 17.93	43.93 ± 19.70	<0.001
**Pre-treatment SATI, categorical**				0.012
< 47.28	207 (65.9%)	66 (80.5%)	141 (60.8%)	
> 47.28	107 (34.1%)	16 (19.5%)	91 (39.2%)	
**SATI change**				0.89
SATI stable (± 10.0%)	108 (34.4%)	27 (32.9%)	81 (34.9%)	
SATI loss (> –10.0%)	90 (28.7%)	23 (28.0%)	67 (28.9%)	
SATI gain (> +10.0%)	116 (36.9%)	32 (39.1%)	84 (36.2%)	
**VATI**	41.61 ± 23.27	37.08 ± 21.80	43.21 ± 23.55	0.04
**Pre-treatment VATI, categorical**				0.005
< 47.54	207 (65.9%)	65 (79.3%)	142 (61.2%)	
> 47.54	107 (34.1%)	17 (20.7%)	90 (38.8%)	
**VATI change**				0.13
VATI stable (± 10.0%)	96 (30.6%)	28 (34.0%)	68 (29.3%)	
VATI loss (> –10.0%)	117 (37.3%)	23 (28.0%)	94 (40.5%)	
VATI gain (> +10.0%)	101 (32.1%)	31 (38.0%)	70 (30.2%)	
**Pre-treatment BMI**	23.36 ± 3.28	21.19 ± 2.81	24.16 ± 3.07	<0.001
**BMI change**				0.23
BMI stable (± 10.0%)	221 (70.4%)	53 (64.6%)	168 (72.4%)	
BMI loss (> –10.0%)	49 (15.6%)	13 (15.9%)	36 (15.5%)	
BMI gain (> +10.0%)	44 (14.0%)	16 (19.5%)	28 (12.1%)	

### Body composition change and postoperative recovery

The association between body composition and perioperative outcomes according to the preoperative SMI category were shown in Table 1. A total of 90 postoperative complications occurred in this study, of which 20 (6.4%) cases of moderate to severe (Clavien-Dindo grade III-V) complications occurred. There were significant differences in frequency of complications between patient groups according to sarcopenia (41.5 vs. 21.4%, *p* = 0.004). The Postoperative LOS (11.21 ± 3.04 vs. 8.92 ± 2.84, *p* < 0.001) was longer in the sarcopenia group than in the non-sarcopenia group, and 30-d readmission (24.4 vs. 6.0%, *p* < 0.001) was higher in the sarcopenia group compared to the non-sarcopenia group. Clinical characteristics and perioperative outcomes according to muscle change are shown in Supplementary Table 1. No significant differences were found between patients according to skeletal muscle loss in terms of postoperative outcomes, including complications, length of postoperative stay and readmission after discharge. Patients with SMI loss seemed to be more likely to have experienced incisional hernia (18.4 vs. 2.6%, *p* < 0.001) after surgery than the patients with non-SMI loss, and the results were statistically significant.

### Body composition change and survival

**FIGURE 2:**
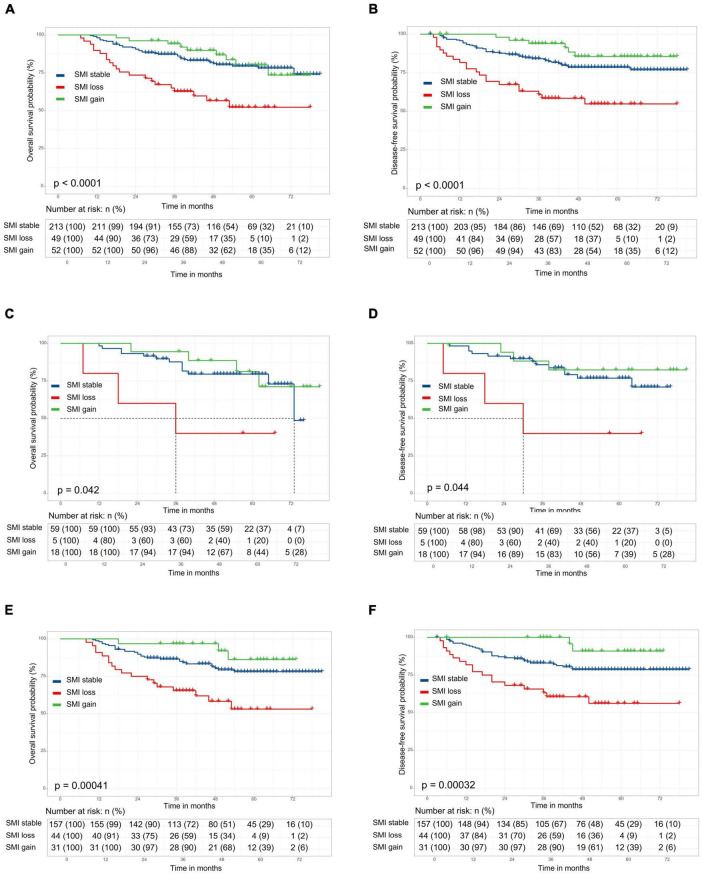
Kaplan–Meier curve demonstrating overall survival and disease-free survival according to skeletal muscle index (SMI) change. The 5-year overall survival (OS) and disease-free survival (DFS) in stocktickerSMI loss, stocktickerSMI stable, and stocktickerSMI gain groups were 52.2, 79.5, and 80.1% [**(A)**, *p* < 0.001] and 54.8, 78.5, and 85.5% [**(B)**, *p* < 0.001], respectively. In a subgroup analysis, patients with stocktickerSMI loss had worse OS and stocktickerDFS in both the preoperative sarcopenia [**(C,D)** and non-sarcopenia groups **(E,F)**].

The length of follow-up ranged from 0.6 to 6.75 years, with a median duration of 3.9 years. The 5-year overall survival (OS) and disease-free survival (DFS) rates for overall patients were 75.4 and 74.8%, respectively. No significant difference in 5-year OS (77.7 vs. 74.7%, respectively; *p* = 0.90) and DFS (72.1 vs. 76.0%, respectively; *p* = 0.99) rate between the preoperative sarcopenia and the non-sarcopenia groups (Supplementary Figures 1A,B). The 5-year OS and DFS in SMI loss, SMI stable and SMI gain groups were 52.2, 79.5, and 80.1% (Figure 2A, *p* < 0.001) and 54.8, 78.5, and 85.5% (Figure 2B, *p* < 0.001), respectively. There were no significant differences in OS and DFS between the two groups according to preoperative SATI (5-year OS: 76.1 vs. 75.1%, *p* = 0.87; 5-year DFS: 74.8 vs. 78.2%, *p* = 0.52) (Supplementary Figures 1C,D). Grouped by preoperative VATI, we found no significant difference in OS (5-year OS: 73.8 vs. 78.6%, *p* = 0.41) and DFS (74.8 vs. 78.1%, *p* = 0.46) (Supplementary Figures 1E,F). In a subgroup analysis, patients with SMI loss had worse OS and DFS in both the preoperative sarcopenia and non-sarcopenia groups (Figures 2C–F). The change in VATI, SATI and BMI were not associated with survival (Supplementary Figure 2).

Skeletal muscle index (SMI) change, TNM stage as risk factors for OS and DFS in the univariate analysis (Supplementary Table 2). After multivariate analysis, SMI change and TNM stage were independently associated with OS (HR: 3.74, 95% CI: 1.96 to 7.12, *p* < 0.001; HR: 3.08, 95% CI: 1.87 to 5.06, *p* < 0.001) and DFS (HR = 3.33; 95% CI, 1.71 to 6.47, *P* < 0.001; HR: 3.05, 95% CI: 1.82 to 5.09, *p* < 0.001) (Table 3). The preoperative BMI, SATI, VATI, and changes in VATI during treatment were not associated with OS or DFS.

**TABLE 3:** Multivariate analysis for overall survival and disease-free survival (*n* = 314).

	Overall survival		Disease-free survival	
	HR and 95% CI	*P*-value	HR and 95% CI	*P*-value
**SMI change**				
SMI stable (± 10.0%)	Reference		Reference	
SMI loss (> –10.0%)	3.74 (1.96–7.12)	<0.001	3.33 (1.71–6.47)	<0.001
SMI gain (> +10.0%)	0.60 (0.27–1.35)	0.22	0.39 (0.16–0.89)	0.06
**TNM stage**				
I–II	Reference		Reference	
III	3.08 (1.87–5.06)	<0.001	3.05 (1.82–5.09)	<0.001
**BMI change**				
BMI stable (± 10.0%)	Reference		Reference	
BMI loss (> –10.0%)	2.04 (1.03–4.05)	0.04	1.72 (0.34–3.52)	0.14
BMI gain (> +10.0%)	1.23 (0.59–2.58)	0.58	1.15 (0.52–2.53)	0.73

## Discussion

In the present study, we demonstrated that skeletal muscle loss negatively impacted oncological outcomes by decreasing OS and DFS in patients with CRC. Patients who had both preoperative sarcopenia and subsequent skeletal muscle loss had the worst OS and DFS. However, preoperative sarcopenia was not a prognostic factor of worse OS and DFS. Which, in line with the recent studies (12, 13), suggesting progressive skeletal muscle loss may be a more potent prognostic factor than a single pre-treatment measurement and highlighting the importance of preserving skeletal muscle mass during treatment in patients with CRC. According to our results, sarcopenic patients were more often readmission after discharge and with a longer length of postoperative LOS than patients without sarcopenia. However, sarcopenia did not increase the rates of Major complications (Clavien Dindo III-V). It suggested that sarcopenia have a negative impact on recovery after colorectal cancer surgery, which is in line with the previous results (14, 15).

In terms of body composition change, we found non-sarcopenic patients were more likely to exhibit skeletal muscle loss during treatment. Because skeletal muscle area changes were used primarily to evaluate skeletal loss in this study, there was a lack of evaluation of skeletal muscle density changes, and in related studies it was also found that patients with sarcopenia were more likely to have decreased skeletal muscle density and fat infiltration. We also found a similar situation in our clinical study. We therefore hypothesize that the reduction in skeletal muscle area precedes the decline in skeletal muscle density and is followed by fatty infiltration. This is an interesting point for subsequent study. Therefore, In addition to advocating skeletal muscle protection for patients with sarcopenia, it is essential to preserve skeletal muscle in patients with non-sarcopenia to minimize the rate of skeletal muscle loss, thereby further blocking the change in skeletal muscle density. Though nutritional intervention combined with physical training programs is broadly accepted as therapeutic options (16) to prevent sarcopenia. The CRC patient’s cohort almost consists of older patients who are not able, sometimes only for a certain period, to be included in physical activity programs. Thus, new pharmaceutical and nutritional interventions and tailor-made physical training for older people need to be explored.

Sarcopenia is considered by most to be an inevitable part of aging. However, the quality and quantity of muscle are dependent upon various factors (17, 18). Such as disease, inactivity, and poor nutrition. Our analysis found no significant differences between patients with SMI loss and non-SMI loss in terms of postoperative outcomes, including complications, length of postoperative stay, and readmission after discharge. Though preoperative sarcopenia has a negative impact on recovery after colorectal cancer surgery, the postoperative skeletal muscle loss does not appear to be related to postoperative recovery in our study. This implies that there are factors other than the postoperative recovery that impact skeletal muscle loss. However, as the interval between pre-and post-treatment CT scans was 6 months, without additional measurements during this interval, it is difficult to assess the exact relationship between postoperative recovery and skeletal muscle loss.

In addition, Ji-Bin Li et al. (19) suggested that a decrease in BMI of more than 5% showed a significantly increased risk of all-cause mortality among CRC patients. In our study, we found the same results. However, we also found that changes in BMI were much less effective in responding to patients’ long-term survival prognosis compared to changes in skeletal muscle, especially with regard to DFS. In the correlation analysis, changes in BMI were moderately correlated with changes in SMI but weakly correlated with changes in SATI and VATI. It may suggest that change in BMI is not sufficiently sensitive to identify clinically meaningful alteration in body composition promptly. There is an emerging viewpoint (20–22) that sarcopenia may be obscured within the bulk of body weight, the patients with identical BMI can have various skeletal muscle. Thus, Body composition quantified using clinically acquired CT images may provide a vital sign to identify patients at increased risk of death.

Higher adiposity increases the risk of colorectal cancer (CRC) and has a negative impact on overall survival (OS) and progression-free survival (PFS) (23, 24). However, in our study, the preoperative SATI and VATI have no association with OS. Although the patients lost SATI and VATI during treatment, the adipose change was not associated with OS and DFS. This discrepancy may be attributed to the small size of our sample, the lack of an optimal cut-off value and the different treatment modalities.

In addition, this study focused on changes in skeletal muscle mass and the absence of the assessment of skeletal muscle strength and function. The lack of consideration of data such as mean skeletal muscle radial decay, patient walking speed, grip strength, and the small sample size are shortcomings of this study. Despite these limitations, the power of this study is that the included cases with detailed treatment and follow-up records, tumors were treated consistently according to clinical treatment guidelines and we conducted a multi-subgroup analysis of the data. Some of our findings are consistent with those of previous studies. Taken together, our results add to the body of evidence linking postoperative skeletal muscle loss to reduced survival.

## Conclusion and implications

In summary, this retrospective study showed that 6-months muscle loss after surgery might affect overall survival and disease-free survival and was an independent predictor of prognosis in patients with CRC. Muscle loss after surgery may be a potential risk factor for incisional hernia. Adequate knowledge of changes in patient body composition by CT during CRC treatment can help clinicians predict outcomes and target nutritional interventions, which may be beneficial in improving the prognosis of CRC patients.

## Data availability statement

The original contributions presented in this study are included in the article/Supplementary material, further inquiries can be directed to the corresponding author/s.

## Ethics statement

The studies involving human participants were reviewed and approved by the Ethics Committee of Xuzhou Central Hospital. The patients/participants provided their written informed consent to participate in this study.

## Author contributions

LZ and LG: conception and design of the study. JG and CD: imaging data analysis. LZ: drafting of the manuscript. LZ and CD: statistical analysis. All authors: acquisition, analysis, or interpretation of data, and critical revision of the manuscript for important intellectual content.

## Supplementary material

Supplementary Figure 1 Kaplan–Meier curve demonstrating overall survival and disease-free survival according to preoperative skeletal muscle index (SMI) **(A,B)**, subcutaneous adiposity index (SATI) **(C,D)**, and visceral adiposity index (VATI) **(E,F)**.

Supplementary Figure 2 Kaplan–Meier curve demonstrating overall survival and disease-free survival according to the change in visceral adiposity index (VATI) **(A,B)**, subcutaneous adiposity index (SATI) **(C,D)**, and body mass index (BMI) **(E,F)**.
