# Supplementary material for: Muscle loss 6 months after surgery predicts poor survival of patients with non-metastatic colorectal cancer
Source: Front Nutr. 2022 Dec 1;9:1047029. doi: 10.3389/fnut.2022.1047029 (PMC9752081; doi:10.3389/fnut.2022.1047029)
Supplement: Supplementary file 1 [file Table_1.docx]

|  | **Overall(n=314)** | | **SMI loss(n=49)** | | **Non-SMI loss (n=265)** | | **p value** | |
| --- | --- | --- | --- | --- | --- | --- | --- | --- |
| **Sex** |  | |  | |  | | 1 | |
| Female | 111 (35.4%) | | 17（34.7%） | | 94（35.5%） | |  | |
| male | 203 (64.6%) | | 32（65.3%） | | 171（64.5%） | |  | |
| **Age** | 58.91±11.48 | | 59.08±11.32 | | 58.88±11.51 | | 0.51 | |
| **CRP(mg/L)** |  | |  | |  | | 0.52 | |
| **>10** | 36(11.5%) | 4(8.0%) | | 32(12.1%) | |  | |  |
| **<10** | 262(83.4%) | | 44(90.0%) | | 218(82.3%) | |  | |
| **Missing** | 16(5.1%) | | 1(2.0%) | | 15(5.6%) | |  | |
| **ALB(g/L)** |  | |  | |  | | <0.001 | |
| **>35** | 301(95.9%) | | 42(85.7%) | | 259(97.7%) | |  | |
| **<35** | 13(4.1%) | | 7(14.3%) | | 6(2.3%) | |  | |
| **Pre-treatment BMI** | 23.36±3.28 | | 24.28±3.49 | | 23.19±3.22 | | 0.21 | |
| **BMI change** |  | |  | |  | | <0.001 | |
| BMI stable (± 10.0%) | 221(70.4%) | | 28(57.2%) | | 193(72.8%) | |  | |
| BMI loss (> –10.0%) | 49(15.6%) | | 18(36.7%) | | 31(11.7%) | |  | |
| BMI gain (> +10.0%) | 44(14.0%) | | 3(6.1%) | | 41(15.5%) | |  | |
| **ASA** |  | |  | |  | | <0.001 | |
| Ⅰ | 178 (56.7%) | | 27（55.1%） | | 151（57.0%） | |  | |
| Ⅱ | 102 (32.5%) | | 15（30.6%） | | 87（32.8%） | |  | |
| Ⅲ | 34 (10.8%) | | 7（14.4%） | | 27（10.2%） | |  | |
| **30-d Any complications** |  | |  | |  | | 0.62 | |
| no | 224 (71.3%) | | 33（67.3%） | | 191(72.1%) | |  | |
| yes | 90 (28.7%) | | 16 (32.7%） | | 74（27.9%） | |  | |
| **30-d Major complications(Clavien Dindo score)** |  | |  | |  | |  | |
| I-Ⅱ | 70 (22.2%) | | 13（26.5%） | | 57（21.5%） | | 1 | |
| Ⅲ-IV | 20 (6.4%) | | 4（8.2%） | | 16（6.0%） | |  | |
| **Operation** |  | |  | |  | | 0.11 | |
| right hemi-colectomy | 28 (8.9%) | | 7（14.3%） | | 21（7.9%） | |  | |
| Left hemi-colectomy | 62 (19.7%) | | 7（14.3%） | | 55（20.8%） | |  | |
| Dixon | 204 (65.0%) | | 32（65.3%） | | 132（49.8%） | |  | |
| Miles | 20 (6.4%) | | 3（6.1%） | | 57（21.5%） | |  | |
| **TNM stage** |  | |  | |  | | 0.17 | |
| Ⅰ | 46 (14.6%) | | 10（20.4%） | | 36（13.6%） | |  | |
| Ⅱ | 150 (47.8%) | | 15（30.6%） | | 135（50.9%） | |  | |
| Ⅲ | 118 (37.6%) | | 24（49%） | | 94（35.5%） | |  | |
| **Neoadjuvant therapy after** |  | |  | |  | | 1 | |
| **preoperative scan** |  | |  | |  | |  | |
| no | 206 (95.1%) | | 34（69.4%） | | 172（64.9%） | |  | |
| yes | 108 (4.9%) | | 15（30.6%） | | 93（35.1%） | |  | |
| **Postoperative LOS, days** | 9.71±2.41 | | 14.42±2.14 | | 11.58±2.39 | | 0.09 | |
| **<=7** | 165 (52.5%) | | 28(56.52%) | | 140(52.89%) | |  | |
| **>7** | 149 (47.5%) | | 21(43.48%) | | 125(47.11%) | |  | |
| **30-d Readmission** |  | |  | |  | |  | |
| no | 280 (89.2%) | | 43(87.8%) | | 237(90.4%) | | 0.92 | |
| yes | 34 (10.8%) | | 6(12.2%) | | 28(10.6%) | |  | |
| **Incisional hernia** |  | |  | |  | | <0.001 | |
| no | 298 (94.9%) | | 40(81.6%) | | 258(97.4%) | |  | |
| yes | 16 (5.1%) | | 9(18.4%) | | 7(2.6%) | |  | |

**Supplementary Table S1** Clinical characteristics and perioperative outcomes according to muscle change (n=314)
