# Supplementary material for: Muscle loss 6 months after surgery predicts poor survival of patients with non-metastatic colorectal cancer
Source: Front Nutr. 2022 Dec 1;9:1047029. doi: 10.3389/fnut.2022.1047029 (PMC9752081; doi:10.3389/fnut.2022.1047029)
Supplement: Supplementary file 2 [file Table_2.docx]

| **Variable** | **Overall survival** | |  | **Disease-free survival** | |  |
| --- | --- | --- | --- | --- | --- | --- |
|  | **Univariate analysis** | |  | **Univariate analysis** | |  |
|  | **Hazard ratio (95% CI)** | ***p*** |  | **Hazard ratio (95% CI)** | ***p*** |  |
| **Age, continuous** | 1.02(1-1.04) | 0.07 |  | 1.02 (0.99-1.04) | 0.06 |  |
| **Smoking** |  |  |  |  |  |  |
| No (never smoked or quitted) | Reference |  |  | Reference |  |  |
| Yes (current smoker) | 1.07(0.80-1.43) | 0.65 |  | 1.01 (0.74-1.36) | 0.98 |  |
| **ASA** |  |  |  |  |  |  |
| I-II | Reference |  |  | Reference |  |  |
| III-IV | 1.12(0.32-2.52) | 0.83 |  | 1.14(0.63-2.05) | 0.67 |  |
| **Pre-treatment BMI, categorical** |  |  |  |  |  |  |
| 18.5-24.9 kg/m^2^ | Reference |  |  | Reference |  |  |
| <18.5 kg/m^2^ | 1.97(0.79-4.96) | 0.15 |  | 1.43 (0.77-2.64) | 0.26 |  |
| ≥25.0 kg/m^2^ | 0.77(0.44-1.35) | 0.36 |  | 1.11 (0.56-2.19) | 0.77 |  |
| **Pre-treatment SMI, categorical** |  |  |  |  |  |  |
| Non-sarcopenia | Reference |  |  | Reference |  |  |
| Sarcopenia | 1.07(0.64-1.79) | 0.81 |  | 0.92 (0.54-1.58) | 0.77 |  |
| **Pre-treatment VATI, categorical** |  |  |  |  |  |  |
| High VATI | Reference |  |  | Reference |  |  |
| Low VATI | 0.99(0.61-1.62) | 0.98 |  | 0.78 (0.48-1.26) | 0.3 |  |
| **Pre-treatment SATI, categorical** |  |  |  |  |  |  |
| High SATI | Reference |  |  | Reference |  |  |
| Low SATI | 1.47(0.88-2.47) | 0.14 |  | 1.30 (0.78-2.17) | 0.32 |  |
| **SMI change, categorical** |  |  |  |  |  |  |
| SMI stable (± 10.0%) | Reference |  |  | Reference |  |  |
| SMI loss (> –10.0%) | 2.75(1.62-4.66) | <0.001 |  | 2.56 (1.50-4.38) | <0.001 |  |
| SMI gain (> +10.0%) | 0.84(0.41-1.73) | 0.64 |  | 0.54 (0.23-1.27) | 0.16 |  |
| **SATI change , categorical** |  |  |  |  |  |  |
| SATI stable (± 10.0%) | Reference |  |  | Reference |  |  |
| SATI loss (> –10.0%) | 1.41(0.76-2.62) | 0.28 |  | 1.05(0.57-1.96) | 0.87 |  |
| SATI gain (> +10.0%) | 1.60(0.90-2.84) | 0.11 |  | 1.21(0.70-2.11) | 0.49 |  |
| **VATI change , categorical** |  |  |  |  |  |  |
| VATI stable (± 10.0%) | Reference |  |  | Reference |  |  |
| VATI loss (> –10.0%) | 1.07(0.62-1.88) | 0.79 |  | 0.97(0.56-1.68) | 0.91 |  |
| VATI gain (> +10.0%) | 1.18(0.46-1.55) | 0.59 |  | 0.72(0.39-1.33) | 0.3 |  |
| **BMI change , categorical** |  |  |  |  |  |  |
| BMI stable (± 10.0%) | Reference |  |  | Reference |  |  |
| BMI loss (> –10.0%) | 1.76(0.98-3.18) | 0.06 |  | 1.43(0.77-2.64) | 0.26 |  |
| BMI gain (> +10.0%) | 1.39(0.95-4.79) | 0.31 |  | 1.11(0.56-2.19) | 0.77 |  |
| **TNM stage** |  |  |  |  |  |  |
| I-II | Reference |  |  | Reference |  |  |
| III | 2.77(1.73-4.45) | <0.001 |  | 2.84 (1.75-4.61) | <0.001 |  |
| **Location of tumour** |  |  |  |  |  |  |
| Right hemicolon | Reference |  |  | Reference |  |  |
| Left hemicolon | 1.35(0.47-3.62) | 0.47 |  | 1.74(0.31-4.09) | 0.61 |  |
| Rectal cancer | 1.57(0.42-3.47) | 0.62 |  | 1.92(0.75-4.37) | 0.14 |  |
| **Postoperative LOS, days** |  |  |  |  |  |  |
| **<=7** | Reference |  |  | Reference |  |  |
| **>7** | 1.20(0.69-2.10) | 0.52 |  | 1.31(0.62-2.46) | 0.42 |  |
| **Hb** |  |  |  |  |  |  |
| ≥ 10 mmol/L | Reference |  |  | Reference |  |  |
| < 10 mmol/L | 1.31 (0.52-2.24) | 0.41 |  | 1.24(0.48-2.01) | 0.64 |  |
| **Neoadjuvant therapy after preoperative scan** |  |  |  |  |  |  |
|  |  |  |  |  |  |  |
| No | Reference |  |  | Reference |  |  |
| Yes | 1.63(0.82-3.26) | 0.17 |  | 2.11(1.08-4.13) | 0.02 |  |
| **30-d Major complications(Clavien Dindo score)** |  |  |  |  |  |  |
| No | Reference |  |  | Reference |  |  |
| Yes | 1.12(0.92-1.36) | 0.26 |  | 1.09(0.88-1.34) | 0.43 |  |

**Supplementary Table S2** Univariable analysis for overall survival and disease-free survival (n=314)
