# Supplementary figures and images for: Muscle loss 6 months after surgery predicts poor survival of patients with non-metastatic colorectal cancer
Source: Front Nutr. 2022 Dec 1;9:1047029. doi: 10.3389/fnut.2022.1047029 (PMC9752081; doi:10.3389/fnut.2022.1047029)

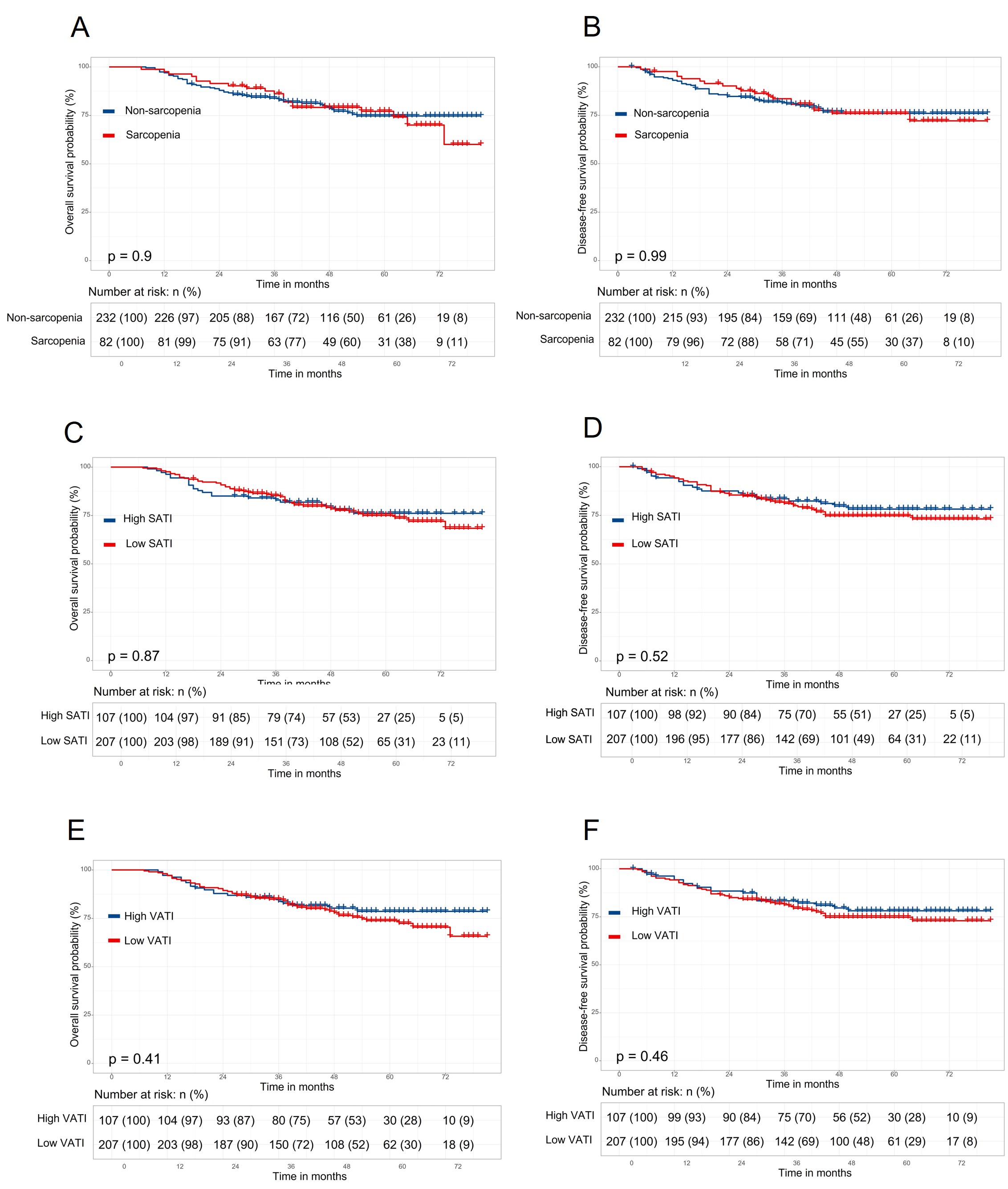

Supplement: Supplementary Figure 1 — Kaplan–Meier curve demonstrating overall survival and disease-free survival according to preoperative skeletal muscle index (SMI) (A,B), subcutaneous adiposity index (SATI) (C,D), and visceral adiposity index (VATI) (E,F). [file Image_1.JPEG]

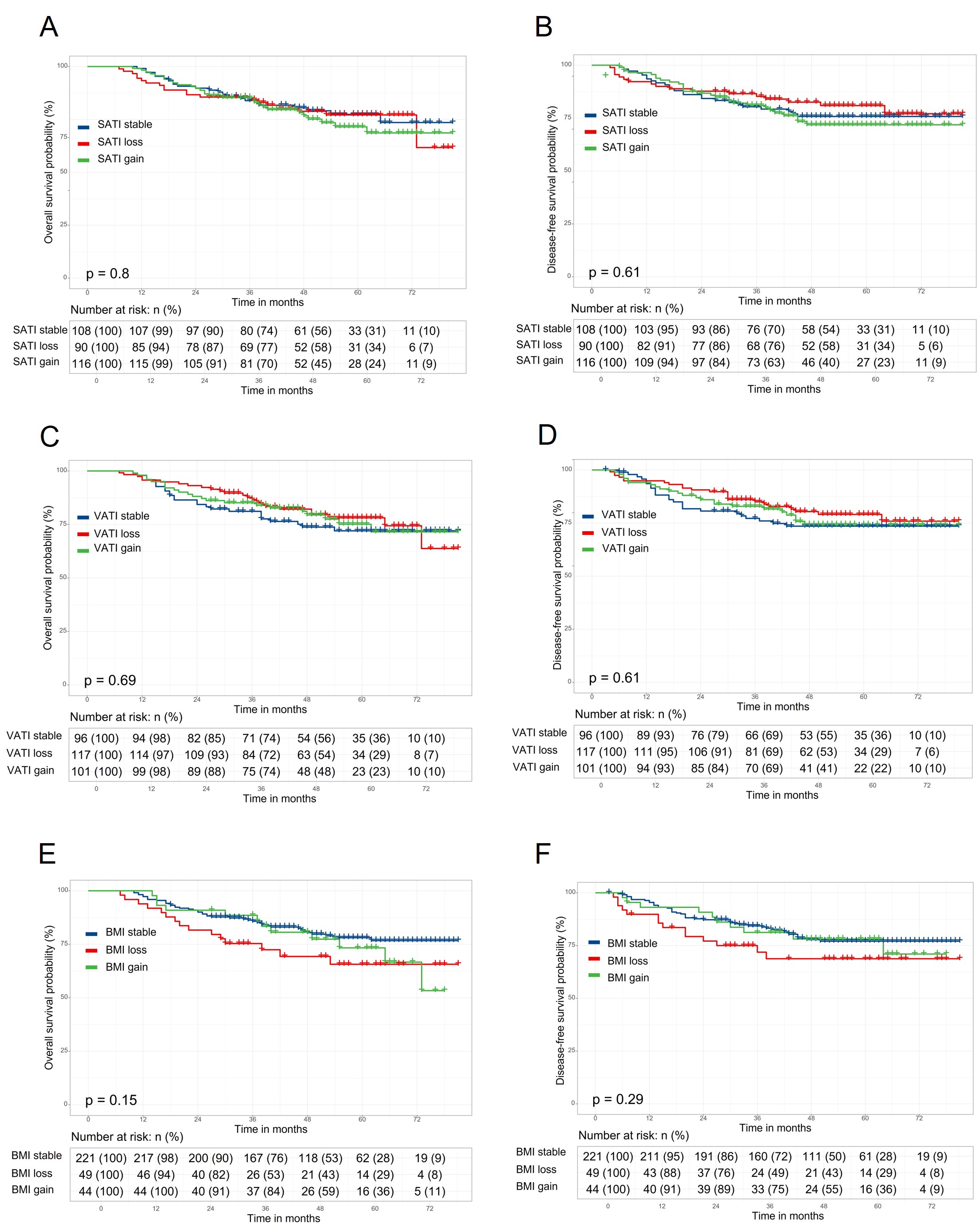

Supplement: Supplementary Figure 2 — Kaplan–Meier curve demonstrating overall survival and disease-free survival according to the change in visceral adiposity index (VATI) (A,B), subcutaneous adiposity index (SATI) (C,D), and body mass index (BMI) (E,F). [file Image_2.JPEG]
